# False negative result of polymerase chain reaction in very early stages of acute retinal necrosis

**DOI:** 10.1186/s12348-023-00366-x

**Published:** 2023-11-08

**Authors:** Haibo Wang, Zhuyun Qian, Lin Cui, Beichen Liu, Jixin Zou, Lu Wang, Yong Tao, Lijun Zhang, Lei Jin

**Affiliations:** 1 Department of Ophthalmology, The Third People’s Hospital of Dalian, Dalian Medical University, Zhongshan District, No. 110, Tianjin Road, Dalian, 116033 China; 2 Beijing GIANTMED Medical Diagnostics Lab, Beijing, China; 3 Department of Ophthalmology, Beijing Chaoyang Hospital, Capital Medical University, Beijing, China; 4 Department of Ophthalmology and Visual Sciences, University of Louisville School of Medicine, Louisville, USA

**Keywords:** Acute retinal necrosis, Polymerase chain reaction, Aqueous humour, False negative result, Case report

## Abstract

**Background:**

Viral nucleic acid testing of intraocular fluid using polymerase chain reaction (PCR) is a major laboratory examination in the diagnosis of acute retinal necrosis (ARN). Importantly, false negative PCR results may occur in several special situations. We reported a case of ARN with a negative PCR result in the aqueous humour in the very early stages of disease.

**Case presentation:**

A female patient presented to the ophthalmologist with complaints of blurred vision and redness in her left eye. Her medical history included ARN in her right eye 10 years prior. Although the result of the aqueous viral analysis by PCR in her left eye was negative the first time (one day after the appearance of ocular symptoms), ARN in her left eye was presumed based on the clinical signs. With timely antiviral and anti-inflammatory treatments, the retinal lesions diminished. The viral load of herpes simplex virus (HSV) turned positive (7.25 × 10^3^ copies/mL) one week later, increased to 2.49 × 10^5^ copies/mL after three weeks, and finally turned negative about five weeks after the onset of disease. The initial HSV-IgG level in the aqueous humour was 0.01 U/mL and increased to 222.64 U/mL in the final sampling.

**Conclusions:**

The results of PCR analysis can be negative in the very early stages of ARN. Diagnosis of ARN should be made based on the clinical features, and antiviral treatments should not be delayed. Repeated PCR analysis of the aqueous humour is necessary to confirm the diagnosis and monitor the disease process.

## Background

Acute retinal necrosis (ARN) is a severe sight-threatening ocular disease characterized by iritis, vitreitis, occlusive retinal vasculitis and necrotizing retinitis. Varicella zoster virus (VZV) and herpes simplex virus (HSV) are two leading causes of ARN. Bilateral involvement of ARN occurs in approximately one-third of patients [1], and the onset of intraocular inflammation in both eyes usually occurs at intervals of several days or weeks [2]. Delayed recurrence of ARN in the second eye, although rare, has been reported to occur up to 46 years later [3]. For a long time, the diagnosis of ARN depended on the clinical findings [4]. With the development of biological technology, analysis of polymerase chain reaction (PCR) and the Goldmann-Witmer coefficient (GWC) were added to further confirm the diagnosis of ARN and to identify the causative virus [5]. These testing methods, usually PCR, are widely used in the diagnosis and treatment of ARN to monitor the activity of the disease [6] and estimate prognosis [7].

In this study, we present a case of ARN in which PCR analysis showed false negative results in the very early stages of disease. Bilateral occurrence of ARN in both eyes with an interval of 10 years occurred in this case, which was uncommon in the clinic.

## Case presentation

A 38-year-old female patient presented to the ophthalmologist on 16 January 2022 with complaints of blurred vision and redness in her left eye for one day. She had suffered sudden visual loss in her right eye 10 years prior, and was diagnosed with ARN in her right eye according to the standard diagnostic criteria for acute retinal necrosis syndrome [4]. No molecular, biological or immunological detection method was applied due to technical limitations at that time. Vitrectomy and silicone oil injections were performed due to rhegmatogenous retinal detachment.

**Fig. 1:**
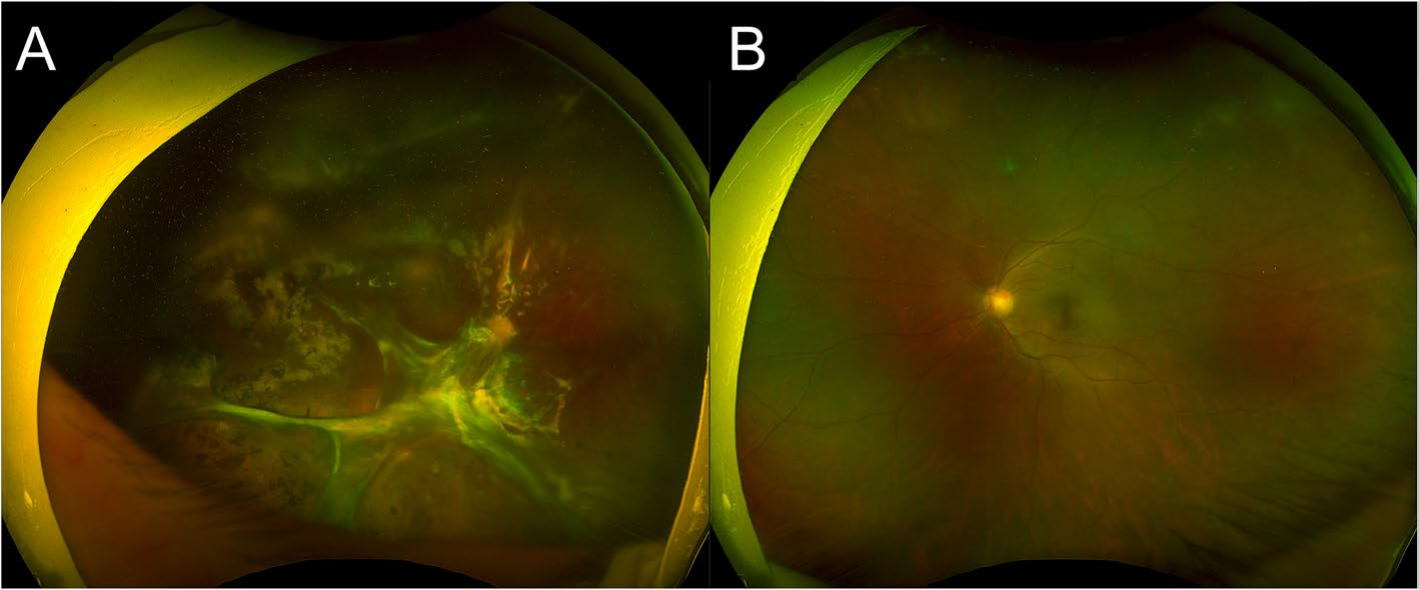
Bilateral ultra-wide field fundus photography taken at the onset of ARN in the left eye. **A** Silicone tamponade in the vitreous cavity, extensive pre-retinal proliferation and retinal scarring could be observed in the right eye. **B** Mild vitritis and peripheral retinal whitening could be observed in the left eye

On initial examination, best-corrected visual acuity (BCVA) was 20/70 (Snellen visual acuity chart) in the left eye and light perception (LP) in the right eye. The intraocular pressure (IOP) was 38 mmHg in the left eye and 17 mmHg in the right eye. Slit-lamp examination revealed conjunctival hyperaemia, mutton-fat keratic precipitates in the middle and below the corneal endothelium, and 3 + flare in the anterior chamber in the left eye. The anterior segment of the right eye was quiet. Dilated fundus examination of the left eye showed mild vitritis, peripheral retinal whitening and mild retinal vasculitis. An exam of the right eye showed silicone tamponade in the vitreous cavity and extensive pre-retinal proliferation (Fig. 1). ARN in the left eye was presumed, and treatments including intravenous acyclovir (1500 mg/day), intravitreal injections of ganciclovir (3 mg) every four days and oral prednisolone were given.

To confirm the diagnosis of ARN and determine the causative virus, about 100μL aqueous humour was obtained through an aqueous tap. Real-time quantitative PCR (qPCR) of four common human herpes viruses (HSV, VZV, CMV, EBV) were performed. For qPCR, a mixture of about 20 μL intraocular fluid and 20 μL nucleic acid extraction liquid (NaCl + EDTA-Na_2_) was incubated at 99 °C for 10 min, then centrifuged at 13,000 rpm for 10 min. The recovered supernatant was used for qPCR assay using HSV (Z-SD-0017–02), VZV (Z-OD-0024–02), CMV (Z-OD-0022–02), and EBV (Z-OD-0023–02-25) real-time PCR kits (Liferiver, Shanghai, China), according to the manufacturer’s instructions. Immunoglobulin G (IgG) measurements of these four viruses were also conducted via enzyme-linked immunosorbent assay (ELISA). Four antibody detection kits, including ESR-105G (HSV1&2-IgG), ESR-104G (VZV-IgG), ESR-109G (CMV-IgG) and ESR-1361G (EB-VCA-IgG) (Institute Virion/Serion GmbH, Würzburg, Germany) were used according to the manufacturer’s instructions.

**Fig. 2:**
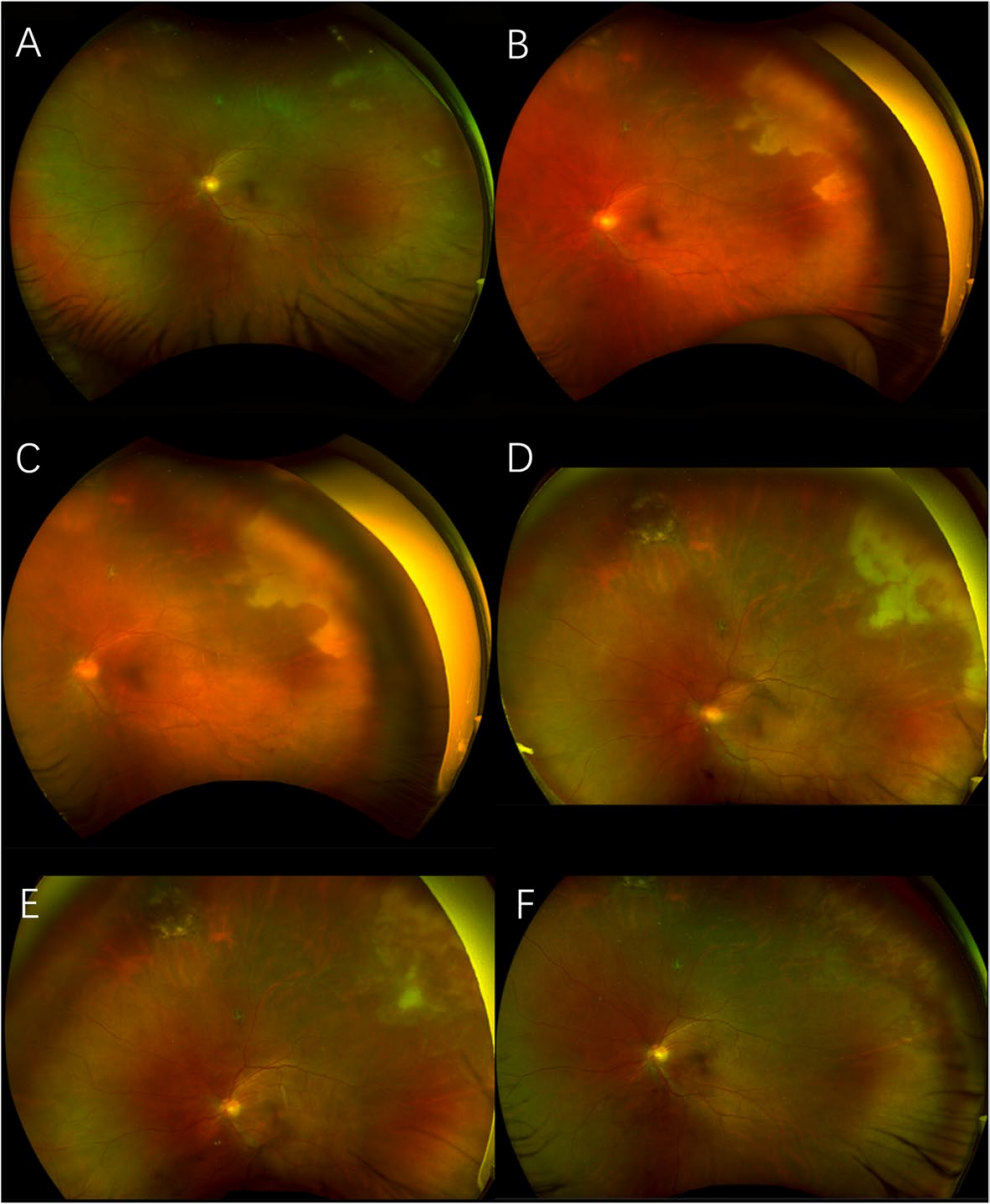
Changes in retinal lesions in the process of ARN. First, the peripheral yellowish-white lesions enlarged quickly towards the posterior pole and then diminished gradually with intensive antiviral and anti-inflammation treatments. **A** Three days after the onset of ARN in the left eye. **B** Ten days after the onset of ARN in the left eye. HSV DNA load in the aqueous humour was 7.25 × 10^3^ copies/mL at this time point. **C** Thirteen days after the onset of ARN in the left eye. **D** About 3 weeks after the onset of ARN in the left eye. HSV DNA load in the aqueous humour was 2.49 × 10^5^ copies/mL at this time point. **E** About 5 weeks after the onset of ARN in the left eye. HSV DNA load in the aqueous humour turned negative at this time point. **F** Thirty-seven days after the onset of ARN in the left eye, which showed nearly complete disappearance of retinal lesions in fundus photography

It was surprising that the results of qPCR for all four viruses were negative. We also did not find any increase in aqueous viral antibody levels. Fundus examination showed an apparent enlargement and integration of retinal lesions towards the posterior pole (Fig. 2). Taking the typical clinical characteristics into consideration, we insisted on the diagnosis of ARN in the left eye and postulated a false negative result from the qPCR. Antiviral treatment was continued. Another aqueous tap was performed on 24 January 2022, about one week after the first sampling, which showed positive HSV DNA load in the aqueous humour (7.25 × 10^3^ copies/mL). Meanwhile, aqueous HSV-IgG was 1.93 U/mL. Despite initial enlargement, the necrotic retinal lesions diminished after intensive antiviral and anti-inflammation treatments (Fig. 2). The HSV DNA load in the aqueous humour was 2.49 × 10^5^ copies/mL on 6 February 2022 and finally turned negative on 17 February 2022, about five weeks after the onset of disease. The level of HSV-IgG in the aqueous humour increased to 276.08 U/mL and 222.64 U/mL at these two time points. BCVA in the left eye was maintained at 20/30. The changes in BCVA, the HSV DNA load and the level of HSV-IgG in the aqueous humour are listed in Table 1.

**Table 1:** The variation of BCVA and the viral load of HSV in the aqueous humour

Date	BCVA (Snellen visual acuity chart)	Aqueous HSV load (copies/mL)	Aqueous HSV-IgG (U/mL)
16.Jan.2022	20/70	(-)	0.01
24.Jan. 2022	20/100	7.25 × 10^3^	1.93
6.Feb.2022	20/70	2.49 × 10^5^	276.08
17.Feb.2022	20/30	(-)	222.64

*BCVA* Best corrected visual acuity, *HSV* Herpes simplex virus

## Discussion and conclusions

PCR analysis of intraocular fluid samples is a useful tool in the diagnosis and monitoring of subsequent treatments for viral posterior uveitis [8–10]. Its high specificity has been verified in previous studies, which have shown that PCR results are negative in most eyes without herpetic uveitis, even in the presence of positive serum antibodies [11]. However, the sensitivity of PCR varies among different studies. Schoenberger et al. reviewed studies with relevance to PCR testing of intraocular fluid samples in the setting of ARN and reported a positive rate of HSV or VZV DNA between 70 and 100% [12]. Several factors can cause a PCR false negative result, including limited amounts of DNA that are lower than required for detection, nucleolysis due to improper sampling or transportation, existence of nucleic acid amplification inhibitor in the reaction system and mutation of the primer binding site [13]. In this case, the most likely reason was the low aqueous HSV DNA load in the very early stages of ARN, which could not be identified by qPCR. The patient had almost no vision in her right eye due to ARN occurring 10 years prior, and used only her left eye in daily life. It was reasonable to deduce that she could easily perceive the loss of vision in her left eye and saw the ophthalmologist earlier than patients with unilateral ARN.

The change in HSV-IgG in the aqueous humour in this case reflected the hose response to the viral infection during disease progression. Errera et al. studied a series of intraocular fluid samples from patients with ARN and proposed that positive PCR results were obtained from 24 h to 10 weeks, but GWC was positive after one week to three months after the onset of signs [14]. In our study, GWC was not calculated because no serum sample was collected. However, it was obvious that the viral antibody in the aqueous humour was maintained at a low level until three weeks after the onset of disease, which indicated that antibody tests in the early stages of ARN should be interpreted with prudence.

Another interesting point in this case was the delayed occurrence of ARN in the second eye after 10 years. The pathogenetic mechanism of bilateral ARN is not fully understood, but the virus is thought to spread via the optic nerve, optic chiasm, parasympathetic pathways and suprachiasmatic nucleus [15–17]. Okunuki et al. [18] reported four cases of delayed occurrence of ARN with latency periods ranging from 3 to 19 years and found that the causative virus species were the same in both eyes. They suggested that the reactivated latent virus that affected the first eye was the causative virus of the disease in the other eye, rather than the second eye being affected by a different virus. It is regretful that the patient in our case did not receive PCR analysis of her intraocular fluid sample 10 years ago, so it was unknown if the causative virus in her two eyes was the same. A previous study also reported less severe inflammation and a lower chance of receiving surgical treatment in the second eye compared to the first affected eye [18], which corresponded to the visual outcome (LP in the right eye and 20/30 in the left eye) and treatment outcome (vitrectomy and silicone oil injections performed in the right eye but not in the left eye) in our case. We propose that the earlier diagnosis and antiviral treatment might contribute to a better prognosis of the eye with later onset.

In conclusion, we reported a case of bilateral ARN with a 10-year interval after the initial onset. The PCR analysis of the aqueous humour did not identify any causative virus the first time, which indicated the false negative possibility of PCR analysis at the onset of ARN. Antibody test results were also low in the early stages of disease. Diagnosis of ARN should be made based on the clinical features, and antiviral treatments should not be delayed. Repeated PCR analysis of the aqueous humour is necessary to further confirm the diagnosis and monitor the disease process.

## Data Availability

All data generated or analyzed during this study are included in this published article.

## Abbreviations

ARN: Acute retinal necrosis
PCR: Polymerase chain reaction
BCVA: Best-corrected visual acuity
IOP: Intraocular pressure
LP: Light perception
HSV: Herpes simplex virus
IgG: Immunoglobulin G
